# Flying After Concussion and Symptom Recovery in College Athletes and Military Cadets

**DOI:** 10.1001/jamanetworkopen.2020.25082

**Published:** 2020-11-11

**Authors:** Tara L. Sharma, Julia Morrow Kerrigan, David L. McArthur, Kevin Bickart, Steven P. Broglio, Thomas W. McAllister, Michael McCrea, Christopher C. Giza

**Affiliations:** 1 Department of Neurosurgery, UCLA Steve Tisch BrainSPORT Program, University of California, Los Angeles; 2 Now with Department of Neurology, University of Washington Medical Center, Seattle; 3 Michigan Concussion Center, University of Michigan, Ann Arbor; 4 Department of Psychiatry, Indiana University School of Medicine, Indianapolis; 5 Department of Neurosurgery and Neurology, Medical College of Wisconsin, Milwaukee; 6 Division of Neurology, Department of Pediatrics, UCLA-Mattel Children’s Hospital, Los Angeles, California

## Abstract

**Importance:**

Concussions are a common occurrence in young athletes. Hypobaric hypoxemia, such as that experienced during airplane travel, can potentially cause alterations to cerebral blood flow and increased neuroinflammatory response. It remains unknown whether flying early after a concussion may influence the clinical course of injury.

**Objective:**

To determine whether there is an association between concussion recovery and airplane travel in collegiate athletes and military cadets.

**Design, Setting, and Participants:**

This cohort study was conducted by the National Collegiate Athletic Association and US Department of Defense Concussion Assessment, Research, and Education Consortium from August 3, 2014, to September 13, 2018. Participant groups were categorized by those who flew within 72 hours of injury and those who did not fly. All participants included in the final analyses had complete data of interest and only 1 injury during the study. Data analysis was performed from September 2018 to March 2020.

**Main Outcomes and Measures:**

Recovery outcome measures were defined as time (in days) from injury to return to activity, school, and baseline symptoms. Symptom and headache severity scores were derived from the Sports Concussion Assessment Tool—Third Edition. Scores for both groups were taken at baseline and a median of 2 days after injury.

**Results:**

A total of 92 participants who flew (mean [SD] age, 19.1 [1.2] years; 55 male [59.8%]) and 1383 participants who did not fly (mean [SD] age, 18.9 [1.3] years; 809 male [58.5%]) were included in the analysis of symptom recovery outcomes (analysis 1). Similarly, 100 participants who flew (mean [SD] age, 19.2 [1.2] years; 63 male [63.0%]) and 1577 participants who did not fly (mean [SD] age, 18.9 [1.3] years; 916 male [58.1%]) were included in the analysis of symptom severity outcomes (analysis 2). No significant group differences were found regarding recovery outcome measures. Likewise, there were no group differences in symptom (estimated mean difference, 0.029; 95% CI, –0.083 to 0.144; *P* = .67) or headache (estimated mean difference, –0.007; 95% CI, –0.094 to 0.081; *P* = .91) severity scores.

**Conclusions and Relevance:**

Airplane travel early after concussion was not associated with recovery or severity of concussion symptoms. These findings may help guide future recommendations on flight travel after concussion in athletes.

## Key Points

**Question:**

Is flying soon after concussion associated with longer recovery or greater symptoms?

**Findings:**

This cohort study found no association between flying and worsened recovery or symptoms after concussion.

**Meaning:**

These findings may provide reassurance to athletic trainers, clinicians, patients, and coaches on the safety of air travel for collegiate athletes and military cadets after concussion.

## Teaser

This cohort study examines whether there is an association between concussion recovery and airplane travel in college athletes and military cadets.

## Introduction

Concussion, a form of mild traumatic brain injury (TBI), is a neurological disturbance following a biomechanical force to the brain and is characterized by a constellation of symptoms including but not limited to headache, dizziness, fatigue, irritability, insomnia, and difficulty with concentration and memory.^1^ Clinical outcome following concussion may be influenced by additional stressors that can affect brain function after initial injury. Some of these stressors include sleep deprivation, pharmaceuticals, psychiatric disorders (eg, anxiety or depression), and dehydration.^2,3^ Hypobaric hypoxemia, such as that encountered during air travel, could also exacerbate concussion-related symptoms and prolong recovery.

Air travel is common among athletes participating in major competitions. Cabin altitude of a pressurized aircraft is maintained at 1520 to 2438 m (5000-8000 feet), which equals an inspired oxygen pressure of 132 to 127 mm Hg compared with 147 mm Hg at sea level.^4,5,6,7^ Exposure to in-cabin pressure at most cruising altitudes has been shown to reduce oxygen saturation levels to below 90% in healthy volunteers.^8,9,10^ One study by Johannigman et al^11^ demonstrated that arterial oxygen desaturation during aeromedical evacuation of military combat casualties at cabin pressure at altitude of 8000 ft (2438 m) occurs frequently, with 90% of patients experiencing at least 1 episode of desaturation to less than 90% during flight. Therefore, passengers traveling on flights can be exposed to reduced oxygen pressure for many hours.^12,13,14,15^ Air travel has also been shown to reduce partial pressure of oxygen and cerebral blood flow in animal models of moderate TBI.^16^ Although hypobaria and hypoxemia during air travel may aggravate moderate to severe disorders, it is currently unclear whether this can exacerbate symptoms after mild TBI.

Studies done in animal models suggest that exposure to aeromedical evacuation at a simulated altitude of 8000 to 8800 ft (2438-2682 m) after mild TBI is associated with decreased arterial oxygen saturation and increased cerebral cytokine expression.^17,18,19^ This neuroinflammatory response, which consists of increased interleukin-6, neuron-specific enolase, and microglial activation, can lead to adverse motor and cognitive consequences.^8,16,17,18,20^ These changes may potentially worsen symptoms and prolong recovery following concussion in humans.

To our knowledge, there are no clinical studies evaluating the effects of airplane travel focused on concussion recovery and severity of symptoms. In this study, we evaluated the effect of airplane travel on postconcussion symptom severity and time to recovery in a large cohort of National Collegiate Athletic Association (NCAA) athletes and military cadets. We hypothesized that flying would exacerbate concussion symptoms and prolong recovery. Knowledge regarding this topic is critical for the development of evidence-based clinical practice recommendations following concussion.

## Methods

### Participants

All study procedures were reviewed and approved by the University of Michigan institutional review board, the US Army Medical Research and Materiel Command Human Research Protection Office, and the local institutional review board at each of the performance sites. Participants provided written informed consent before participation. This study follows the Strengthening the Reporting of Observational Studies in Epidemiology (STROBE) reporting guideline.

This study analyzed data from a prospective observational cohort obtained from the Concussion Assessment, Research, and Education Consortium database. In this database, an average of 90% of eligible participants, which included any NCAA varsity athlete or military service academy cadet, completed baseline data across 29 locations. The data set provided for this study included only the 3480 NCAA athletes and military service academy cadets who experienced a concussion from August 3, 2014, to September 13, 2018. Military service cadets were athletes involved in either NCAA-level sports (NCAA athletes) or intramural or club sports (non-NCAA athletes). We combined both military cadets and NCAA athletes because both groups have high incidence of concussion and share similar physical and demographic characteristics.^21^

All concussions were diagnosed using the following definition: “a change in brain function following a force to the head, which may be accompanied by temporary loss of consciousness, but is identified in awake individuals with measures of neurologic and cognitive dysfunction.”^21,22^ Identification, assessment, and diagnosis of concussive events were completed by the medical staff at each site.^21^ From this cohort, we selected participants who had a defined flying status, including 165 who flew and 2235 who did not fly. We only included participants who flew within 72 hours of injury to capture participants who flew soon after injury without substantially decreasing our sample size. This was also a time point used in some animal studies of simulated aeromedical evacuation.^18,19^

Participants were excluded if they had missing data of interest and more than 1 injury during the study (eFigure 1 and eFigure 2 in the Supplement). Two separate cohorts were created for each analysis, symptom recovery (analysis 1) and symptom severity (analysis 2), to include more participants who flew with complete demographic and injury information. Demographic variables chosen are factors associated with prolonged concussion recovery and, thus, are potential confounders if they are not comparable between groups. These include age,^23,24^ sex,^25,26,27,28^ sport type,^29^ concussion history,^30,31,32^ preexisting nonmigraine and migraine headaches,^33,34^ and depression.^30,33^ We reported injury characteristics that are known to be associated with recovery, including delayed reporting of symptoms, loss of consciousness, and posttraumatic amnesia. Delayed reporting of symptoms was recorded as the time between the onset of postconcussive symptoms and the time these symptoms were reported to medical staff. We also compared demographic and injury characteristics between each analysis group and the original population to confirm that groups were similar and selection bias was minimal. All site personnel were trained on a standardized protocol for baseline testing and postinjury assessments before data collection and participant written informed consent were obtained.^22^

### Symptom Recovery Outcomes

The Concussion Assessment, Research, and Education data set includes a broad range of demographic, injury, and outcome variables. We selected variables that have been previously used in published studies and were collected on the greatest number of participants.^33^ These variables represent clinical recovery and return to activity and school. These included number of days after injury until starting a graded return to play protocol (RTP start), returning to learn in full school (RTL), and symptom resolution (SR). SR was defined as the number of days after the injury when concussion-related symptoms returned to the preinjury state (ie, baseline symptom severity on Sport Concussion Assessment Tool—Third Edition [SCAT3]).

### Symptom and Headache Severity Outcomes

Information for total symptom and headache severity secondary to concussion was derived from the SCAT3. Clinical assessments included measures of concussion symptoms (SCAT3). The SCAT3 includes a 22-item self-report inventory designed to evaluate the total number and severity of common concussion symptoms on a 7-point Likert scale where 0 indicates none and 6 indicates severe. Total symptom severity was evaluated using the total severity score from the SCAT3 (22 items × 6 levels gives a range of 0-132). Headache severity from the SCAT3 was determined by the 7-point Likert scale (range, 0-6).^35^ We evaluated headache severity separately because this is a common concussion-related symptom that is experienced in some individuals even without concussion during flight.^36^ We analyzed these SCAT3 scores reported at baseline (before the start of the athletic season) and after injury. For participants who flew, we analyzed postinjury SCAT3 symptom and headache severity scores taken after flight.

### Statistical Analysis

#### Between-Group Analysis

The χ^2^ test was used for categorical data, and the independent *t* test was used for continuous data to compare baseline demographics variables between groups. For *t* tests, degrees of freedom were corrected when the Levene test indicated inequality of variance between groups. Medians and interquartile ranges (IQRs) were reported for days after injury until SCAT3 evaluation, time from injury to flight, and duration of flight given the skewed distribution of the data. Analyses used the general linear model, which incorporates both continuous and discrete variables, to assess the effect of airplane travel on selected outcome measures together with covariates (see the eAppendix in the Supplement for statistical software commands and outputs). Because not reporting a concussion immediately after injury has been associated with longer symptom recovery, we adjusted for delayed symptom reporting as described already in the Participant subsection of the Methods.^37,38^ We also adjusted for sport category (contact, limited contact, noncontact, and non-NCAA athlete),^39^ baseline SCAT3 symptom and headache severity raw scores, and number of days after injury until SCAT3 evaluation. All outcome variables and covariates were logarithmically transformed given the nonnormal distribution of the data, and 0.1 was added to all values before logarithmic transformation to avoid nonsignificant results for entries of 0. Tukey honestly significantly different test was used post hoc to determine estimated mean difference between groups.

#### Within-Group Analysis

An analysis of variance was used to determine whether there was an association between the number of time zones crossed (ie, 0, 1, 2, 3, or 4) and logarithmically transformed symptom recovery and severity variables. This is important because crossing multiple time zones may result in sleep deprivation, fatigue, and mood issues, which can potentially worsen concussion symptoms and prolong recovery.^40^ The analysis of variance was recalculated to rescore 1 participant who flew across more than 3 time zones. This made the 3–time zone category 3 or more time zones (ie, 0, 1, 2, and ≥3). Partial eta-squared η_p_^2^ was used to estimate effect size (small, 0.01; medium, 0.09; large, 0.25). A Spearman ρ test was used to determine whether the number of hours of plane travel and time from injury to flight correlated with the symptom recovery and severity variables. To control for sex, sport type, and overrepresentation of football players in the concussed cohort (288 participants [19.6%] in analysis 1 and 368 participants [21.9%] in analysis 2; eTable 1 in the Supplement) we repeated the between group analysis including only football players.

#### Conditions for All Analyses

We accepted a 2-sided *P* < .05 as significant with Bonferroni correction for multiple comparisons. All analyses were conducted using SPSS statistical software version 25 (IBM) and R statistical software version 3.6.3 (R Project for Statistical Computing). Data analysis was performed from September 2018 to March 2020.

## Results

### Descriptive Analysis

A total of 92 participants who flew (mean [SD] age, 19.1 [1.2] years; 55 male [59.8%]) and 1383 participants who did not fly (mean [SD] age, 18.9 [1.3] years; 809 male [58.5%]) were included in the analysis of symptom recovery outcomes (analysis 1). Similarly, 100 participants who flew (mean [SD] age, 19.2 [1.2] years; 63 male [63.0%]) and 1577 participants who did not fly (mean [SD] age, 18.9 [1.3] years; 916 male [58.1%]) were included in the analysis of symptom severity outcomes (analysis 2).

Analyzed cohorts were similar to the original population except for number of days after injury concussion symptoms were reported late and percentage of non-NCAA level athletes (eTable 2 in the Supplement). Groups (flew vs did not fly) were similar, with no significant differences in demographic variables except for history of depression and percentage of non-NCAA athletes (Table 1). For NCAA level athletes, numbers of participants for each sport category and type are presented in eTable 1 in the Supplement. The median (IQR) time from injury to flight was 12 (4-30) hours. The median (IQR) flight time was 2 (1.5-3) hours. The median (IQR) time after injury until SCAT3 evaluation was 2 (1.75-3) days for participants who flew and 2 (1-4) days for participants who did not fly. Frequency distributions of logarithmically transformed symptom recovery and symptom and headache severity outcome variables (at postinjury and baseline time points) were conducted and are presented in eFigure 3 and eFigure 4 in the Supplement. Participants who recovered shortly after injury were documented as 0 days and thus presented as negative log values (eFigure 3 in the Supplement).

**Table 1.** Demographic Variables and Injury Characteristics for Analyses 1 and 2 in All Participants Who Flew and Did Not Fly

Variable	Participants, No. (%)					
	Analysis 1			Analysis 2		
	Flew (n = 92)	Did not fly (n = 1383)	*P* value	Flew (n = 100)	Did not fly (n = 1577)	*P* value
Sex						
Male	55 (59.8)	809 (58.5)	.81	63 (63.0)	916 (58.1)	.33
Female	37 (39.8)	574 (41.5)		37 (37.0)	661 (41.9)	
Age, mean (SD), y	19.1 (1.2)	18.9 (1.3)	.80	19.2 (1.2)	18.9 (1.3)	.53
NCAA athlete^a^						
Collision or contact	54 (74.0)	643 (69.4)	.42	62 (56.7)	736 (68.3)	.25
Limited contact	10 (13.7)	185 (20.0)		11 (15.1)	223 (20.7)	
Noncontact	9 (12.3)	98 (10.6)		8 (9.2)	119 (11.0)	
Non-NCAA athlete^a^	19 (20.7)	457 (33.0)	.01	19 (19.0)	499 (31.6)	.008
Injury characteristics						
Amnesia	8 (8.7)	141 (10.2)	.64	12 (12.0)	156 (9.9)	.50
Loss of consciousness	6 (6.2)	93 (6.7)	.94	6 (6.0)	108 (6.8)	.74
Days symptoms reported late	1.8 (2.4)	1.5 (2.4)	.44	0.51 (1.3)	0.94 (2.3)	.06
History of concussion	38 (43.2)	462 (34.7)	.11	43 (43.0)	541 (34.3)	.08
History of migraine headaches	9 (10.2)	88 (6.6)	.19	10 (10.0)	103 (6.5)	.18
History of nonmigraine headache	1 (1.1)	20 (1.5)	.78	3 (3.0)	26 (1.6)	.32
History of headaches in the past 3 mo	25 (28.7)	491 (36.9)	.08	32 (32.7)	558 (37.3)	.35
History of depression	6 (6.8)	35 (2.6)	.02	8 (8.0)	39 (2.5)	.001
SCAT3 total symptom severity baseline scores	NA	NA	NA	5.6 (9.1)	6.5 (10.9)	.41
SCAT3 headache severity baseline scores	NA	NA	NA	0.3 (0.8)	0.4 (0.9)	.51

Abbreviations: NA, not applicable; NCAA, National Collegiate Athletic Association; SCAT3, Sports Concussion Assessment Tool 3.

^a^ NCAA athletes were either collegiate athletes or military cadets who played NCAA sports. Non-NCAA athletes were military cadets who did not participate in NCAA sports. Sport level of contact was only obtained for those playing NCAA sports.

### Overall Cohort Analysis

#### Between-Group Analysis

**Figure 1.**
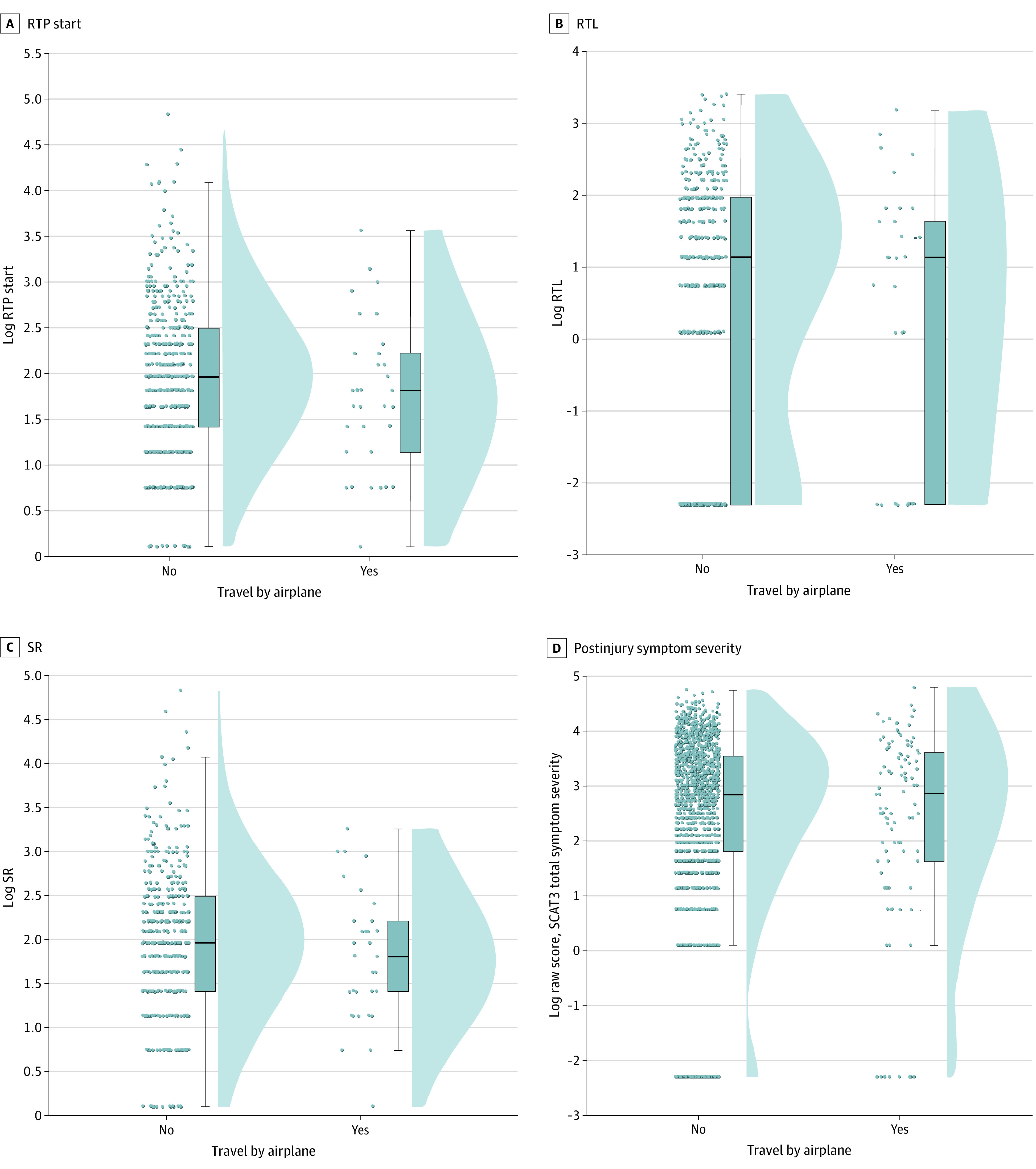
Raincloud Plots for All Participants Who Flew and Did Not Fly Evaluating for Symptom Recovery and Symptom Severity Outcome Variables Panel A shows the return to play (RTP) start, which is the number of days after injury for participants to start a graded RTP protocol. Panel B shows the return to learn (RTL), which is the number of days after injury to RTL in full school. Panel C shows the number of days after injury for concussion-related symptoms to return to the preinjury state (symptom resolution [SR]). Panel D shows the postinjury symptom severity. For each box plot, the horizontal line inside the box indicates the median, boundaries of the box indicate the 25th and 75th percentiles, and whiskers indicate the minimum and maximum values. Scatter plots to the left and density plots to the right show the individual values and distribution of values, respectively. Values were logarithmically transformed given skewness in distribution. SCAT3 indicates Sports Concussion Assessment Tool 3.

There was no significant difference between groups in terms of RTP start (flew vs did not fly estimated mean difference, –0.016; 95% CI, –0.072 to 0.040; *P* = .68), RTL (flew vs did not fly estimated mean difference, 0.087; 95% CI, –0.026 to 0.200; *P* = .30), and SR (flew vs did not fly estimated mean difference, –0.046; 95% CI, –0.115 to 0.023; *P* = .23) (see Figure 1A-C and coefficients tables for parameter estimates in eAppendix in the Supplement). There was no significant difference between groups in terms of symptom severity (flew vs did not fly estimated mean difference, 0.029; 95% CI, –0.083 to 0.144; *P* = .67) and headache severity (flew vs did not fly estimated mean difference, –0.007; 95% CI, –0.094 to 0.081; *P* = .91) (see Figure 1D and coefficients table for parameter estimates in the eAppendix in the Supplement).

#### Within-Group Analysis

There was no association between time zones crossed and symptom recovery variables or symptom and headache severity scores (Table 2 and eFigure 5 in the Supplement). There was also no difference in total hours traveled and symptom recovery variables or symptom and headache severity scores (Spearman ρ range = –0.061 to 0.099; *P* = .37-.81). There was also no significant difference in time from injury to flight and symptom recovery variables or symptom and headache severity scores (Spearman ρ range = 0.069-0.152; *P* = .14-.51) (eFigure 6 in the Supplement).

**Table 2.** Comparison of Outcome Variables and Number of Time Zones Crossed During Air Travel

Variables^a^	Mean (SD), time zones crossed				ANOVA		Effect size, η_p_^2^^b^
	0	1	2	3	*F*	*P* value	
Return to play start^c^	0.8 (0.4)	0.8 (0.4)	1.0 (0.3)	0.8 (0.5)	0.601_3,90_	.62	.021
Return to learn^d^	0.2 (0.9)	0.5 (0.8)	0.7 (0.6)	−0.2 (1.1)	2.40_3,90_	.07	.078
Symptom resolution^e^	0.8 (0.6)	0.8 (0.4)	0.9 (0.4)	0.7 (0.5)	0.277_3,90_	.84	.010
SCAT3 total symptom severity raw scores^f^	0.9 (0.9)	0.8 (0.9)	1.0 (0.4)	0.9 (0.7)	0.161_3,70_	.92	.007
SCAT3 headache severity raw scores^f^	0.1 (0.6)	−0.1(0.7)	0.1 (0.4)	0.1 (0.7)	0.607_3,70_	.61	.027

Abbreviations: ANOVA, analysis of variance; SCAT3, Sports Concussion Assessment Tool 3.

^a^ All values were logarithmically transformed given the skewness in distribution.

^b^ η_p_^2^ is measured as partial eta squared estimates.

^c^ Refers to number of days after injury for participants to start a graded return to play protocol.

^d^ Refers to number of days after injury to return to learn in full school.

^e^ Refers to number of days after injury for concussion-related symptoms to return to preinjury state. For symptom recovery variables, n = 90.

^f^ For total symptom and headache severity scores, n = 70.

### Football-Only Analysis

**Figure 2.**
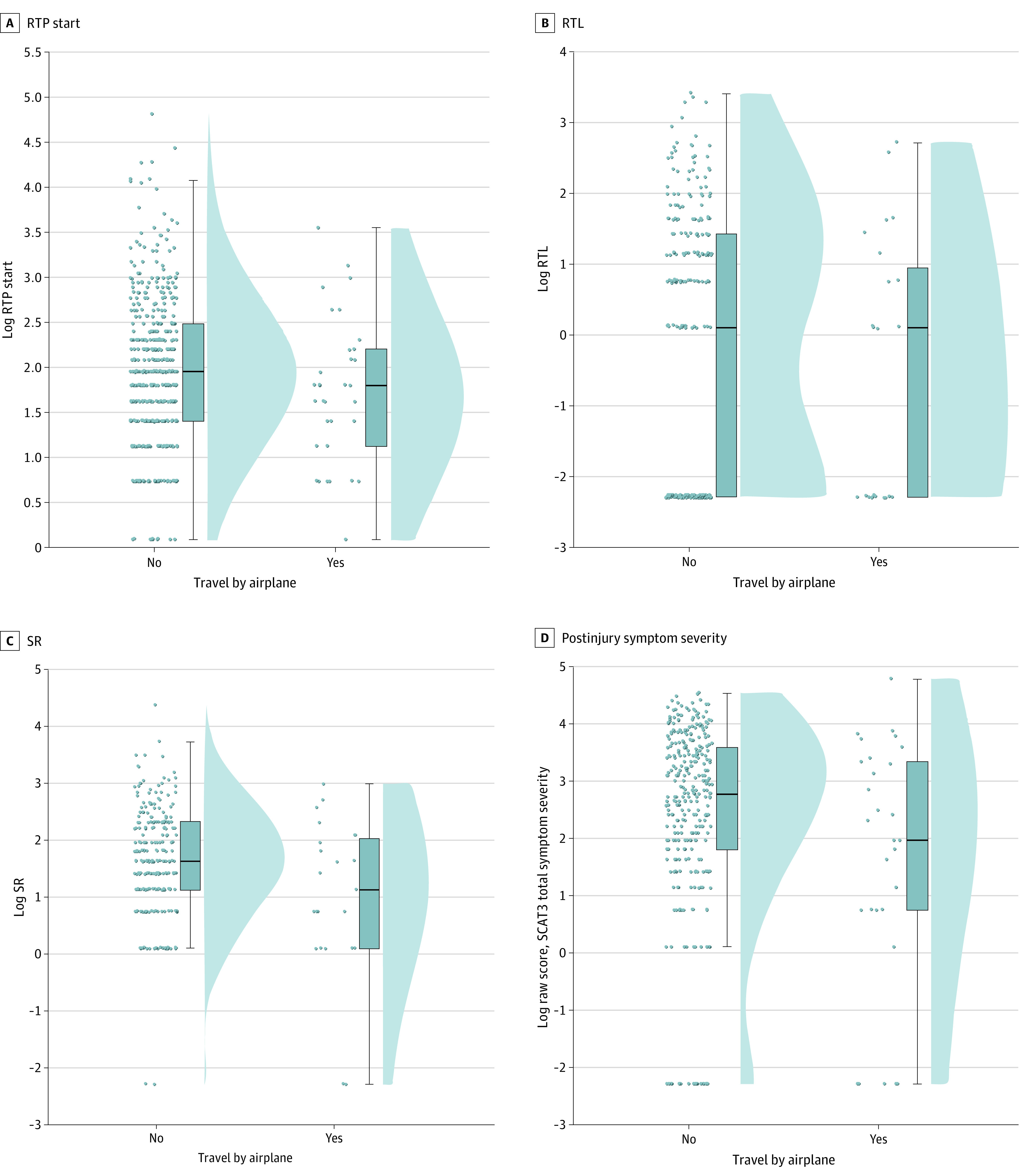
Raincloud Plots for Only Football Players Who Flew and Did Not Fly Evaluating for Symptom Recovery and Symptom Severity Outcome Variables Panel A shows the return to play (RTP) start, which is the number of days after injury for participants to start a graded RTP protocol. Panel B shows the return to learn (RTL), which is the number of days after injury to RTL in full school. Panel C shows the number of days after injury for concussion-related symptoms to return to the preinjury state (symptom resolution [SR]). Panel D shows the postinjury symptom severity. For each box plot, the horizontal line inside the box indicates the median, boundaries of the box indicate the 25th and 75th percentiles, and whiskers indicate the minimum and maximum values. Scatter plots to the left and density plots to the right show the individual values and distribution of values, respectively. Values were logarithmically transformed given skewness in distribution. SCAT3 indicates Sports Concussion Assessment Tool 3.

When looking at football players only, the group of participants who flew were similar to the group of participants who did not fly, showing no significant difference in demographic variables (see Table 3 for demographic characteristics, group sizes, and comparison). There were no significant differences between groups in RTP start (flew vs did not fly estimated mean difference, –0.088; 95% CI, –0.192 to 0.016; *P* = .24), RTL (flew vs did not fly estimated mean difference, –0.118; 95% CI, –0.335 to 0.098; *P* = .53), and SR (flew vs did not fly estimated mean difference, –0.256; 95% CI, –0.432 to –0.080; *P* = .08) (Figure 2A-C). There was also no significant difference between groups in symptom severity (flew vs did not fly estimated mean difference, –0.309; 95% CI, –0.579 to –0.039; *P* = .16) (Figure 2D) and headache severity (flew vs did not fly estimated mean difference, –0.260; 95% CI, –0.449 to –0.070; *P* = .12) scores between groups.

**Table 3.** Demographic Variables for Analysis 1 and 2 in Football Players Who Flew and Did Not Fly

Variable	Athletes, No. (%)					
	Analysis 1			Analysis 2		
	Flew (n = 23)	Did not fly (n = 265)	*P* value	Flew (n = 29)	Did not fly (n = 339)	*P* value
Age, mean (SD), y	19.2 (1.2)	19.0 (1.3)	0.50	19.3 (1.1)	18.9 (1.3)	.11
Injury characteristics						
Loss of consciousness	3 (13.0)	19 (7.2)	.31	3 (10.3)	16 (4.7)	.19
Amnesia	3 (13.0)	24 (9.1)	.53	5 (17.2)	50 (14.7)	.72
Days symptoms reported late	0.65 (1.9)	0.59 (1.5)	.84	0.28 (0.70)	0.64 (1.5)	.20
History of concussion	13 (56.5)	108 (40.8)	.14	14 (48.3)	145 (42.8)	.57
History of migraine headache	2 (8.7)	22 (8.3)	.94	1 (3.4)	31 (9.1)	.30
History of nonmigraine headache	0 (0.0)	6 (2.3)	.47	1 (3.4)	6 (1.8)	.53
History of headaches in the past 3 mo	7 (30.4)	78 (29.4)	.91	10 (34.5)	109 (32.2)	.80
History of depression	0 (0.0)	5 (1.9)	.517	1 (3.4)	6 (1.8)	.53
SCAT3 total symptom severity baseline scores	NA	NA	NA	3.6 (4.9)	4.6 (9.8)	.61
SCAT3 headache severity baseline scores	NA	NA	NA	0.28 (0.70)	0.28 (0.78)	.99

Abbreviations: NA, not applicable; SCAT3, Sports Concussion Assessment Tool 3.

## Discussion

Air travel is thought to worsen long-term neurological outcomes of mild TBI, but evidence supporting this association is currently unclear.^8,17,18^ In this study, we found no association between air travel and prolonged recovery or increased symptom severity after concussion. The number of time zones crossed during flight was not associated with any of our outcome variables. These findings were similar in both the entire cohort and the subset of football players. This is consistent with a study published in 1961,^41^ which found that symptoms related to flight travel were minimal in 47 military cadets with varying degrees of brain injuries, along with other injuries, who traveled in a modern pressurized aircraft. Taken together with our results, this suggests that air travel may not be associated with longer recovery or greater symptoms following sports-related concussion.

One possible explanation is that pressurized aircraft minimize the physiological problems caused by low ambient pressure and high altitude such as hypoxia. For example, one study by Muhm et al^42^ showed that exposure to hypobaric conditions, like that experienced during commercial aircraft flights, lowered oxygen saturation in passengers by 4%. However, this relative decrease in oxygen saturation was insufficient to cause acute mountain sickness, adverse health outcomes, or impairment in sensory or psychomotor performance.^42^ Other studies have shown that neurological deficits induced by hypoxia are not apparent in healthy individuals at altitudes below 10 000 ft (3048 m).^43^ The risks for negative neurological outcomes from lower inspired oxygen are further reduced by the sedentary nature (ie, sitting) of air travel.^5^ Furthermore, there is currently limited evidence showing how moderate reductions in oxygen saturation during air travel can lead to worsening long-term clinical outcomes.^12^

Another possible explanation is that healthy, fit individuals such as athletes are more likely to acclimatize to the physiological changes induced by air travel. For example, one study^12^ showed no significant change in pulse rates in athletes who flew. The authors postulated that these fit individuals may have adaptive mechanisms for facilitating oxygen transport in hypoxic conditions, resulting in a smaller compensatory effect on the cardiovascular system.^12^ This physiological adaptation may also result in clinically insignificant symptoms during air travel.

There is evidence that hypobaric conditions, like those during air travel, might lead to temporary worsening of symptoms in participants with a history of concussion. For example, one study^44^ evaluating neurocognitive outcomes at a simulated altitude of 3800 m in participants with history of concussion demonstrated cognitive impairment, particularly on memory tasks, immediately after exposure. However, neurocognitive function returned to baseline as soon as the hypobaric conditions were removed. This indicates that hypobaric conditions may exacerbate symptoms only temporarily. Our study brings this finding a step further by showing that concussed athletes may not experience long-term neurological dysfunction after air travel.

In terms of pathophysiology, an augmented neuroinflammatory response may depend on time after injury to hypobaric exposure. Specifically, increased neuroinflammatory markers were found in mice exposed to hypobaric conditions 3 hours after mild TBI, but delayed exposure (24 hours after injury) showed no deleterious effects.^8^ Therefore, although we found no differences in concussion recovery or symptoms related to air travel, there may be changes at a molecular level in athletes who flew closer to their injury. Future studies with molecular biomarkers would be needed to address this question.

### Limitations

There are limitations to this study. One is that a limited number of athletes flew within a few hours of their concussion. There were also very few athletes for whom time to SCAT3 evaluation was within a few hours of a flight. Because of this, we could not adequately determine how flying can acutely affect severity of concussion symptoms. Whether the concussion occurred outside of school session (ie, during breaks or holidays) was also not reported. This could affect our recovery variables, particularly RTL. Furthermore, the median number of hours traveled by airplane was only 2, with a small number of athletes crossing 3 or more time zones. As a result, we could not ascertain whether long-haul flights, in which people are exposed to reduced oxygen pressures for a longer amount of time, influence the course of recovery from concussion.^5,12^

## Conclusions

Overall, this cohort study found that athletes flying within 72 hours of concussion did not show greater symptoms, prolonged symptom recovery, or delayed return to activity or school. These findings may be reassuring to patients, coaches, and athletic trainers. Further studies should aim to investigate certain predisposing factors that may prolong recovery after flight along with a higher powered analysis of earlier flight times (ie, flight times within 6 hours of injury), specific sports, and duration of flight. This may help to more accurately determine the optimal time to fly following concussion or mild TBI. Taken together, we conclude that flying is not associated with continued neurological dysfunction after concussion.

## Notes

Supplement. **eFigure 1.** Subjects Who Met Inclusion/Exclusion Criteria for Analysis 1 **eFigure 2. **Subjects Who Met Inclusion/Exclusion Criteria for Analysis 2 **eTable 1.** Sport Category and Type for All NCAA Level Athletes **eTable 2.** Demographic Comparisons Between Subjects Included in Both the Symptom Recovery (Analysis 1) and Severity Analyses (Analysis 2) and Original Study Population **eAppendix.** Statistical Mixed Effects Model Analysis Output for Overall Cohort Analyses **eFigure 3.** Frequency Distribution of Symptom Recovery Outcomes **eFigure 4.** Frequency Distribution of Logarithmically Transformed Symptom and Headache Severity Outcomes at Baseline and Post Injury **eFigure 5. **Comparison of Symptom Recovery and Symptom Severity Outcome Variables With Number of Time Zones Crossed During Air Travel **eFigure 6.** Comparison of Symptom Recovery and Severity Outcome Variables and Time From Injury to Flight
