# Supplementary material for: Flying After Concussion and Symptom Recovery in College Athletes and Military Cadets
Source: JAMA Netw Open. 2020 Nov 11;3(11):e2025082. doi: 10.1001/jamanetworkopen.2020.25082 (PMC7658735; doi:10.1001/jamanetworkopen.2020.25082)
Supplement: Supplement. — eFigure 1. Subjects Who Met Inclusion/Exclusion Criteria for Analysis 1 eFigure 2. Subjects Who Met Inclusion/Exclusion Criteria for Analysis 2 eTable 1. Sport Category and Type for All NCAA Level Athletes eTable 2. Demographic Comparisons Between Subjects Included in Both the Symptom Recovery (Analysis 1) and Severity Analyses (Analysis 2) and Original Study Population eAppendix. Statistical Mixed Effects Model Analysis Output for Overall Cohort Analyses eFigure 3. Frequency Distribution of Symptom Recovery Outcomes eFigure 4. Frequency Distribution of Logarithmically Transformed Symptom and Headache Severity Outcomes at Baseline and Post Injury eFigure 5. Comparison of Symptom Recovery and Symptom Severity Outcome Variables With Number of Time Zones Crossed During Air Travel eFigure 6. Comparison of Symptom Recovery and Severity Outcome Variables and Time From Injury to Flight [file jamanetwopen-e2025082-s001.pdf]

Supplemental Online Content

Sharma TL, Morrow Kerrigan J, McArthur DL, et al; CARE Consortium Investigators. Flying after concussion and symptom recovery in college athletes and military cadets. *JAMA Netw Open*. 2020;3(11):e2025082. doi:10.1001/jamanetworkopen.2020.25082

eFigure 1. Subjects Who Met Inclusion/Exclusion Criteria for Analysis 1

eFigure 2. Subjects Who Met Inclusion/Exclusion Criteria for Analysis 2

eTable 1. Sport Category and Type for All NCAA Level Athletes

eTable 2. Demographic Comparisons Between Subjects Included in Both the Symptom Recovery (Analysis 1) and Severity Analyses (Analysis 2) and Original Study Population

eAppendix. Statistical Mixed Effects Model Analysis Output for Overall Cohort Analyses

eFigure 3. Frequency Distribution of Symptom Recovery Outcomes

eFigure 4. Frequency Distribution of Logarithmically Transformed Symptom and Headache Severity Outcomes at Baseline and Post Injury

eFigure 5. Comparison of Symptom Recovery and Symptom Severity Outcome Variables With Number of Time Zones Crossed During Air Travel

eFigure 6. Comparison of Symptom Recovery and Severity Outcome Variables and Time From Injury to Flight

This supplemental material has been provided by the authors to give readers additional information about their work.

© 2020 Sharma TL et al. *JAMA Network Open.*

eFigure 1. Subjects Who Met Inclusion/Exclusion Criteria for Analysis 1

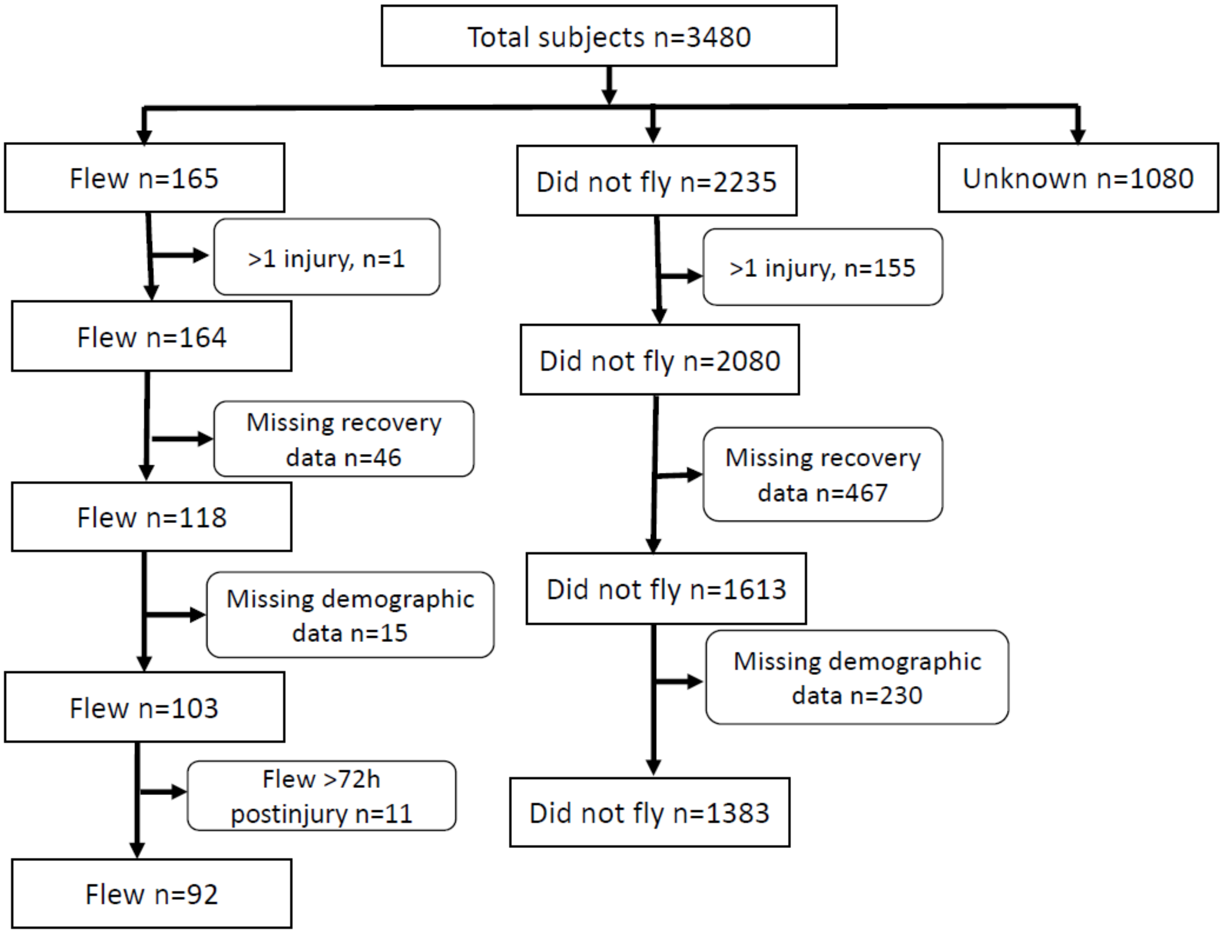

© 2020 Sharma TL et al. *JAMA Network Open.*

eFigure 2. Subjects Who Met Inclusion/Exclusion Criteria for Analysis 2

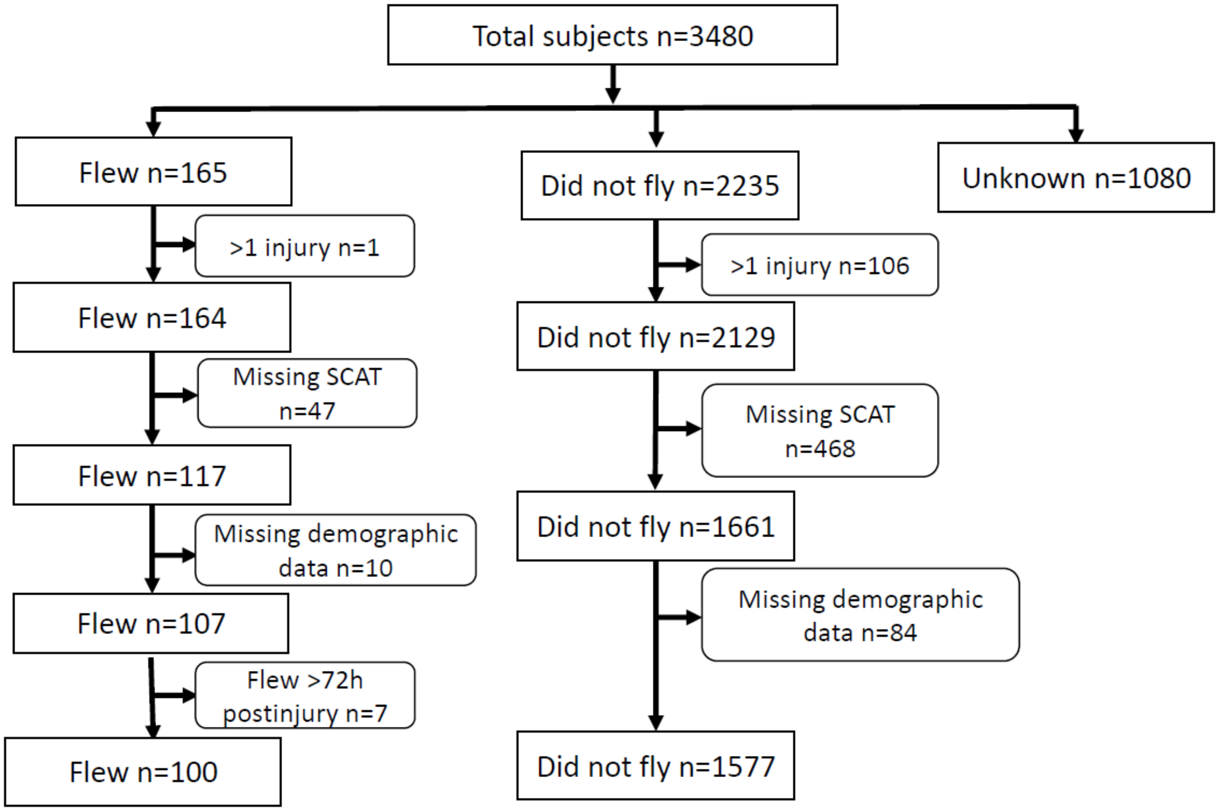

© 2020 Sharma TL et al. *JAMA Network Open.*

eTable 1. Sport Category and Type for All NCAA Level Athletes

	Analysis 1		Analysis 2	
**Sport Category**	**Flew (n=73)**	**Did not fly (n=926)**	**Flew (n=81)**	**Did not Fly (n=1078)**
**Contact**				
Football	23	265	29	339
Basketball	2	70	1	66
Diving	1	16	1	17
Ice Hockey	1	15	2	18
Lacrosse	8	58	9	56
Soccer	7	154	6	160
Water Polo	5	21	6	27
Wrestling	4	30	5	36
Field Hockey	3	14	3	17
**Limited Contact**				
Baseball	2	24	1	33
Gymnastics	1	29	1	33
Beach Volleyball	0	4	0	4
Volleyball	1	56	4	67
Fencing	0	5	0	5
Cross Country/Track	5	22	3	27
Softball	0	34	1	42
Field Event	1	11	1	12

© 2020 Sharma TL et al. *JAMA Network Open.*

	**Analysis 1**		**Analysis 2**	
**Sport Category**	**Flew (n=73)**	**Did not Fly (n=926)**	**Flew (n=81)**	**Did not Fly (n=1078)**
**Non-Contact**				
Tennis	3	16	3	18
Cheerleading	2	38	1	41
Golf	1	1	1	1
Rifle	1	3	1	3
Rowing/Crew	1	11	1	12
Swimming	1	29	1	44

All values are presented as number of subjects.

© 2020 Sharma TL et al. *JAMA Network Open.*

eTable 2. Demographic Comparisons Between Subjects Included in Both the Symptom Recovery (Analysis 1) and Severity Analyses (Analysis 2) and Original Study Population

	**Original Population (n=3480)**	**Analysis 1 (n=1475)**	**P value**	**Analysis 2 (n=1677)**	**P value**
Sex (%)					
Male	2081 (59.8)	864 (56.8)	0.423	979 (58.4)	0.331
Female	1399 (40.2)	611 (39.4)		698 (41.6)	
Age (Years)[a]	19.0 (1.4)	19.0 (1.2)	0.990	18.9 (1.3)	0.117
NCAA Athlete (%)[b]					
Collision/Contact	1844 (53.0)	697 (47.3)	0.482	798 (47.6)	0.183
Limited Contact	466 (13.4)	195 (13.2)		234 (14.0)	
Non-Contact	258 (7.4)	107 (7.3)		127 (7.6)	
Non-NCAA Athlete (%)[b]	912 (26.2)	476 (32.3)	**0.001**	518 (30.9)	**0.001**
**Injury Characteristics**					
Amnesia	368 (10.6)	149 (10.1)	0.648	168 (10.0)	0.592
LOC	228 (6.60)	99 (6.71)	0.836	114 (6.78)	0.765
Days symptoms reported late	1.7 (2.9)	1.23 (1.7)	**0.001**	0.91 (2.2)	**0.001**
History of Concussion (%)	1286 (37.0)	513 (34.8)	0.146	584 (34.8)	0.138
History of Non-migraine headaches	60 (1.7)	21 (1.4)	0.540	29 (1.7)	0.990
History of Migraine Headaches (%)	211 (6.1)	97 (6.6)	0.520	113 (6.7)	0.358
History of Depression	116 (3.3)	41 (2.7)	0.330	47 (2.8)	0.350

© 2020 Sharma TL et al. *JAMA Network Open.*

eAppendix. Statistical Mixed Effects Model Analysis Output for Overall Cohort Analyses

```
> dir()[9]
[1] "FLY and NO Fly after concussion recovery data FINAL.xlsx"
## R version 3.6.3
## require:
## readit, readxl, sjmisc, ggplot2, cowplot, dplyr
> df <- as.data.frame(readit(dir()[9]))                    ## read and subset data frame
> s1 <- subset(df,select=c(35,36,37,8,28,42))
> s1 <- remove.missing(s1)                                 ## drop cases with missing values
> for (i in 1:3)s1[,i] <- num(s1[,i])                      ## change chr to num as needed
> s1$log.rtpdaysofconcussionsymptoms <- log(s1$rtpdaysofconcussionsymptoms+0.1) ## calculate log
## variables each augmented by 0.1
> s1$log.acaperform <- log(s1$acaperform+0.1)              ## to render original zeros useful
> s1$log.gradexertstart <- log(s1$gradexertstart+0.1)
> s1$log.daysreportedlate <- log(s1$daysreportedlate+0.1)
> s1$trav<- substr(s1$travelbyairplane,1,1)
>
> stru(s1)
[1] 0 rtpdaysofconcussionsymptoms : num 1 8 5 5 30 4 3 7 11 14 ...
[2] 0 acaperform : num 0 0 5 1 30 0 0 0 0 0 ...
[3] 0 gradexertstart : num 3 6 6 9 45 4 4 7 11 11 ...
[4] 0 travelbyairplane : chr "Yes" "Yes" "Yes" "Yes" ...
[5] 0 daysreportedlate : num 0 2 0 2 0 4 0 1 3 11 ...
[6] 0 sporttype : chr "Contact Sport" "Contact Sport" "Non-Contact spor
[7] 0 log.rtpdaysofconcussionsymptoms: num 0.0953 2.0919 1.6292 1.6292 3.4045 ...
[8] 0 log.acaperform : num -2.3026 -2.3026 1.6292 0.0953 3.4045 ...
[9] 0 log.gradexertstart : num 1.13 1.81 1.81 2.21 3.81 ...
[10] 0 log.daysreportedlate : num -2.303 0.742 -2.303 0.742 -2.303 ...
-=- data.frame -=- 1475 obs. of 10 variables -=-
> stats(s1$log.rtpdaysofconcussionsymptoms,by=s1$travelbyairplane)          ## log ( SR )
by travel
          No  Yes
N 1383.0000 92.0000
mean 1.9505 1.7874
SD 0.9221 1.1229
robust min 0.0953 0.0953
min -2.3026 -2.3026
hdQ:0.25 1.4105 1.1538
median 1.9779 1.9345
hdQ:0.75 2.5594 2.6082
max 5.5876 3.5863
robust max 4.1912 3.5863
skew -0.3001 -1.0928
kurtosis 1.0086 2.2064
> summary(glm(log.rtpdaysofconcussionsymptoms ~ travelbyairplane + log.daysreportedlate + sporttype,
data=s1 ))

Deviance Residuals:
Min 1Q Median 3Q Max
-4.0503 -0.4875 0.0530 0.5745 3.5650
Coefficients:
Estimate Std. Error t value Pr(>|t|)
```

© 2020 Sharma TL et al. *JAMA Network Open.*

```
(Intercept) 1.98431 0.04115 48.224 < 2e-16 ***
travelbyairplaneYes -0.10677 0.09386 -1.138 0.255477
log.daysreportedlate 0.17723 0.01581 11.211 < 2e-16 ***
sporttypeLimited Contact sport 0.17152 0.07049 2.433 0.015088 *
sporttypeNon-Contact sport 0.31847 0.09005 3.537 0.000418 ***
sporttypeNon-NCAA athlete 0.44636 0.05251 8.501 < 2e-16 ***
---
Signif. codes: 0 '***' 0.001 '**' 0.01 '*' 0.05 '.' 0.1 ' ' 1
```

Pg 2

```
(Dispersion parameter for gaussian family taken to be 0.755735)
Null deviance: 1292.1 on 1474 degrees of freedom
Residual deviance: 1110.2 on 1469 degrees of freedom
AIC: 3780.8
Number of Fisher Scoring iterations: 2
> stats(s1$log.acaperform,by=s1$travelbyairplane)        ## log ( RTL)
by travel
No Yes
N 1383.0000 92.0000
mean 0.5964 0.6858
SD 1.8458 1.9329
robust min -2.3026 -2.3026
min -2.3026 -2.3026
hdQ:0.25 -1.0690 -1.1090
median 1.0788 1.1326
hdQ:0.75 1.9741 2.1375
max 4.7458 3.5863
robust max 4.7458 3.5863
skew -0.5594 -0.5255
kurtosis -0.9827 -1.0266
> summary(glm(log.acaperform ~ travelbyairplane + log.daysreportedlate + sporttype, data=s1 ))
Deviance Residuals:
Min 1Q Median 3Q Max
-3.6514 -1.2364 0.4798 1.3461 4.5470
Coefficients:
Estimate Std. Error t value Pr(>|t|)
(Intercept) 0.25601 0.08526 3.003 0.002722 **
travelbyairplaneYes 0.20024 0.19448 1.030 0.303380
log.daysreportedlate 0.06433 0.03276 1.964 0.049750 *
sporttypeLimited Contact sport 0.52219 0.14607 3.575 0.000362 ***
sporttypeNon-Contact sport 0.67944 0.18659 3.641 0.000281 ***
sporttypeNon-NCAA athlete 0.92263 0.10880 8.480 < 2e-16 ***
---
Signif. codes: 0 '***' 0.001 '**' 0.01 '*' 0.05 '.' 0.1 ' ' 1
(Dispersion parameter for gaussian family taken to be 3.245001)
Null deviance: 5049.4 on 1474 degrees of freedom
Residual deviance: 4766.9 on 1469 degrees of freedom
AIC: 5930.1
```

Pg 3

```
> stats(s1$log.gradexertstart,by=s1$travelbyairplane)        ## log ( RTP
start ) by travel
     No      Yes
N  1383.0000 92.0000
mean 1.9982 1.8983
```

© 2020 Sharma TL et al. *JAMA Network Open.*

```
SD 0.8712 0.9359
robust min 0.0953 0.0953
min -2.3026 0.0953
hdQ:0.25 1.4112 1.2036
median 1.9978 1.8441
hdQ:0.75 2.5773 2.6386
max 4.8291 3.8733
robust max 4.2918 3.8733
skew -0.0754 0.0528
kurtosis 0.1550 -0.6503
> summary(glm(log.gradexertstart ~ travelbyairplane + log.daysreportedlate + sporttype, data=s1 ))
Deviance Residuals:
Min 1Q Median 3Q Max
-4.4362 -0.4907 0.0071 0.5380 2.7254
Coefficients:
Estimate Std. Error t value Pr(>|t|)
(Intercept) 1.98637 0.03809 52.146 < 2e-16 ***
travelbyairplaneYes -0.03584 0.08689 -0.413 0.680016
log.daysreportedlate 0.15819 0.01463 10.810 < 2e-16 ***
sporttypeLimited Contact sport 0.17349 0.06526 2.658 0.007935 **
sporttypeNon-Contact sport 0.31146 0.08336 3.736 0.000194 ***
sporttypeNon-NCAA athlete 0.51154 0.04861 10.524 < 2e-16 ***
---
Signif. codes: 0 ‘***’ 0.001 ‘**’ 0.01 ‘*’ 0.05 ‘.’ 0.1 ‘ ’ 1
(Dispersion parameter for gaussian family taken to be 0.6476879)
Null deviance: 1129.61 on 1474 degrees of freedom
Residual deviance: 951.45 on 1469 degrees of freedom
AIC: 3553.2
> s2 <- subset(df,select=c(14,50,43,35,24,16))                ## read and subset data frame
> s2 <- remove.missing(s2)                                     ## drop cases with missing values
> for (i in c(1,3,5,6) s2[,i] <- num(s2[,i)                    ## change chr to num as needed
> s2$log.scatsexsevscorepostinjury <- log(s2[,1]+0.1)          ## calculate log variables each
                                                               ## augmented by 0.1
> s2$log.daysreportedlate <- log(s2[,3]+0.1)                   ## to render original zeros useful
> s2$log.scatsexsevscorebaseline <- log(s2[,5]+0.1)
> s2$log.timetoscat <- log(s2[,6]+0.1)
> stru(s2)
[1] 0 scatsxsevscorepostinjury : num 26 45 33 5 38 19 6 44 3 53 ...
[2] 0 travelbyairplane : chr "Yes" "Yes" "Yes" "Yes" ...
[3] 0 daysreportedlate : num 0 3 0 0 1 1 0 0 5 0 ...
[4] 518 sporttype : chr "Limited Contact sport" "Contact sport" "Contact sp
[5] 0 scatsxsevscorebaseline : num 4 17 12 4 1 13 3 10 0 0 ...
[6] 0 timetoscat : num 2 4 1 12 2 1 1 1 5 2 ...
[7] 0 log.scatsexsevscorepostinjury: num 3.26 3.81 3.5 1.63 3.64 ...
[8] 0 log.daysreportedlate : num -2.3026 1.1314 -2.3026 -2.3026 0.0953 ...
[9] 0 log.scatsexsevscorebaseline : num 1.411 2.8391 2.4932 1.411 0.0953 ...
[10] 0 log.timetoscat : num 0.7419 1.411 0.0953 2.4932 0.7419 ...
-=- data.frame -=- 1677 obs. of 10 variables -=-
Pg 4
> stats(s2$log.scatsexsevscorepostinjury, by=s2$travelbyairplane)
No Yes
N 1577.0000 100.0000
mean 2.4446 2.3624
```

© 2020 Sharma TL et al. *JAMA Network Open.*

SD 1.5747 1.7645
robust min 0.0953 0.0953
min -2.3026 -2.3026
hdQ:0.25 1.8120 1.6075
median 2.8295 2.8377
hdQ:0.75 3.5210 3.6212
max 4.7458 4.7883
robust max 4.7458 4.7883
skew -1.5254 -1.3907
kurtosis 2.3341 1.5125
> summary(glm(log.scatsexsevscorepostinjury ~ travelbyairplane + log.daysreportedlate + sporttype + log.scatsexsevscorebaseline + log.timetoscat, data=s2))
Deviance Residuals:
Min 1Q Median 3Q Max
-5.1249 -0.6461 0.3135 1.0753 2.9326
Coefficients:
Estimate Std. Error t value Pr(>|t|)
(Intercept) 3.13970 0.10267 30.581 < 2e-16 ***
travelbyairplaneYes 0.07985 0.18473 0.432 0.66564
log.daysreportedlate 0.27151 0.03635 7.470 1.58e-13 ***
sporttypeLimited Contact sport 0.37277 0.11947 3.120 0.00185 **
sporttypeNon-Contact sport 0.62039 0.15350 4.042 5.66e-05 ***
log.scatsexsevscorebaseline 0.03012 0.02299 1.310 0.19042
log.timetoscat -0.69696 0.06869 -10.146 < 2e-16 ***

Signif. codes: 0 ‘***’ 0.001 ‘**’ 0.01 ‘*’ 0.05 ‘.’ 0.1 ‘ ’ 1
(Dispersion parameter for gaussian family taken to be 2.551022)
Null deviance: 3311.2 on 1158 degrees of freedom
Residual deviance: 2938.8 on 1152 degrees of freedom
(518 observations deleted due to missingness)
AIC: 4383.5

© 2020 Sharma TL et al. *JAMA Network Open.*

eFigure 3 Frequency Distribution of Symptom Recovery Outcomes

Outcomes were logarithmically transformed to reduce skewness in the distribution (A: RTP start, B: RTL and C: SR).
Abbreviations: RTP Start: number of days after injury for subjects to start a graded return to play protocol, RTL: number of days to return to learn in full school, SR: number of days after injury for concussion-related symptoms to return to preinjury state.

A.

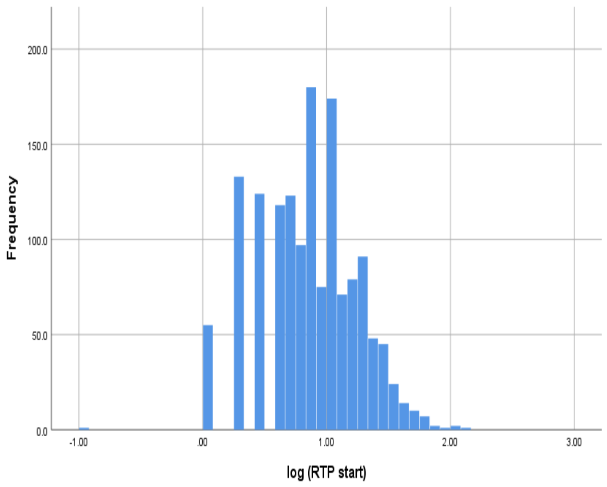

B.

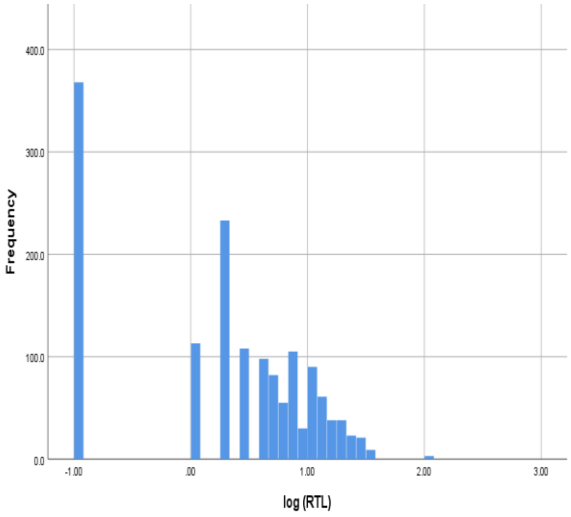

C.

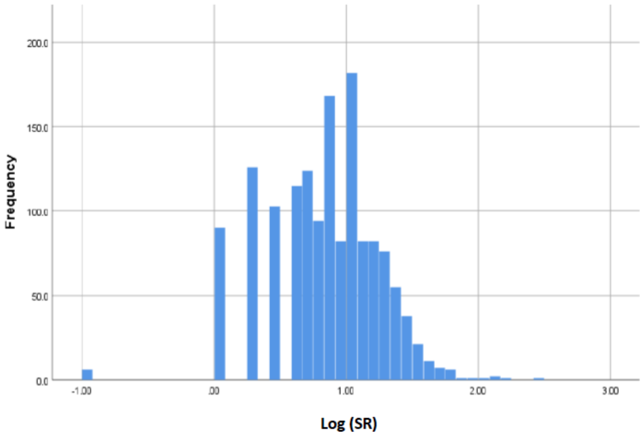

© 2020 Sharma TL et al. *JAMA Network Open.*

eFigure 4. Frequency Distribution of Logarithmically Transformed Symptom and Headache Severity Outcomes at Baseline and Post Injury

SCAT3 raw scores were logarithmically transformed to reduce skewness in the distribution.

A. Symptom severity post-injury

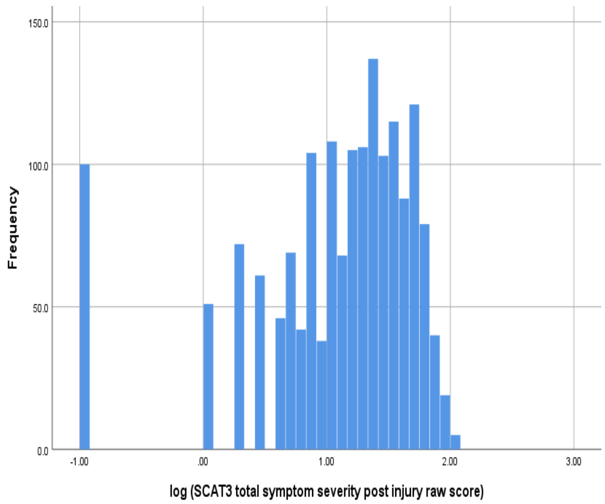

B. Symptom severity baseline

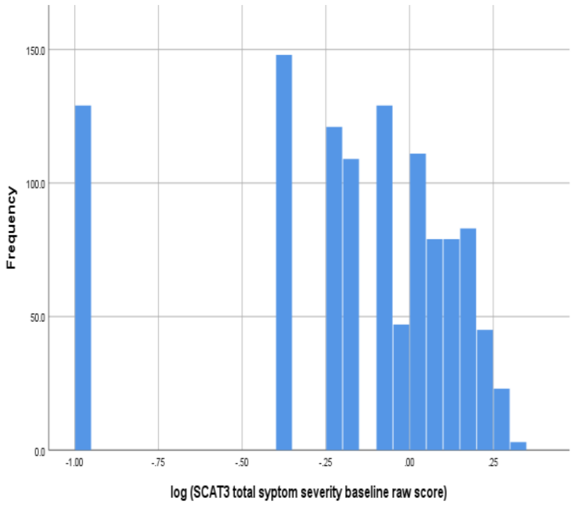

C. Headache severity post-injury

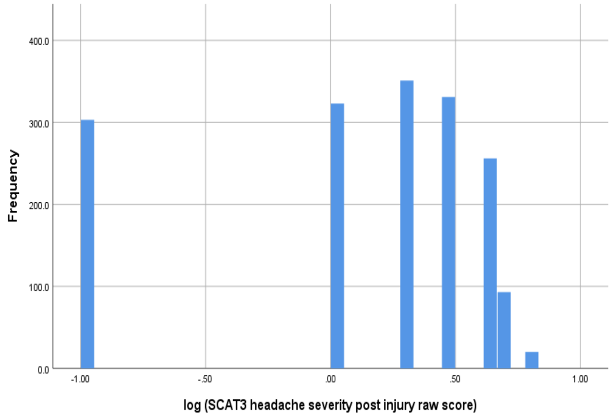

D. Headache severity baseline

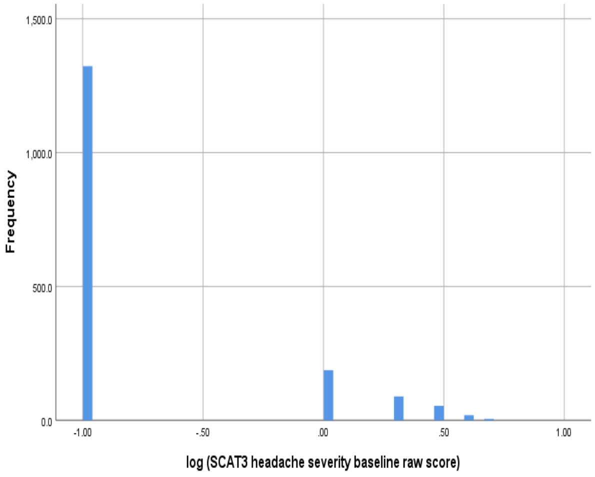

© 2020 Sharma TL et al. *JAMA Network Open.*

eFigure 5. Comparison of Symptom Recovery and Symptom Severity Outcome Variables With Number of Time Zones Crossed During Air Travel

See eFigure 3 for abbreviations (A: RTP start, B: RTL, C: SR, D: Symptom severity post-injury)

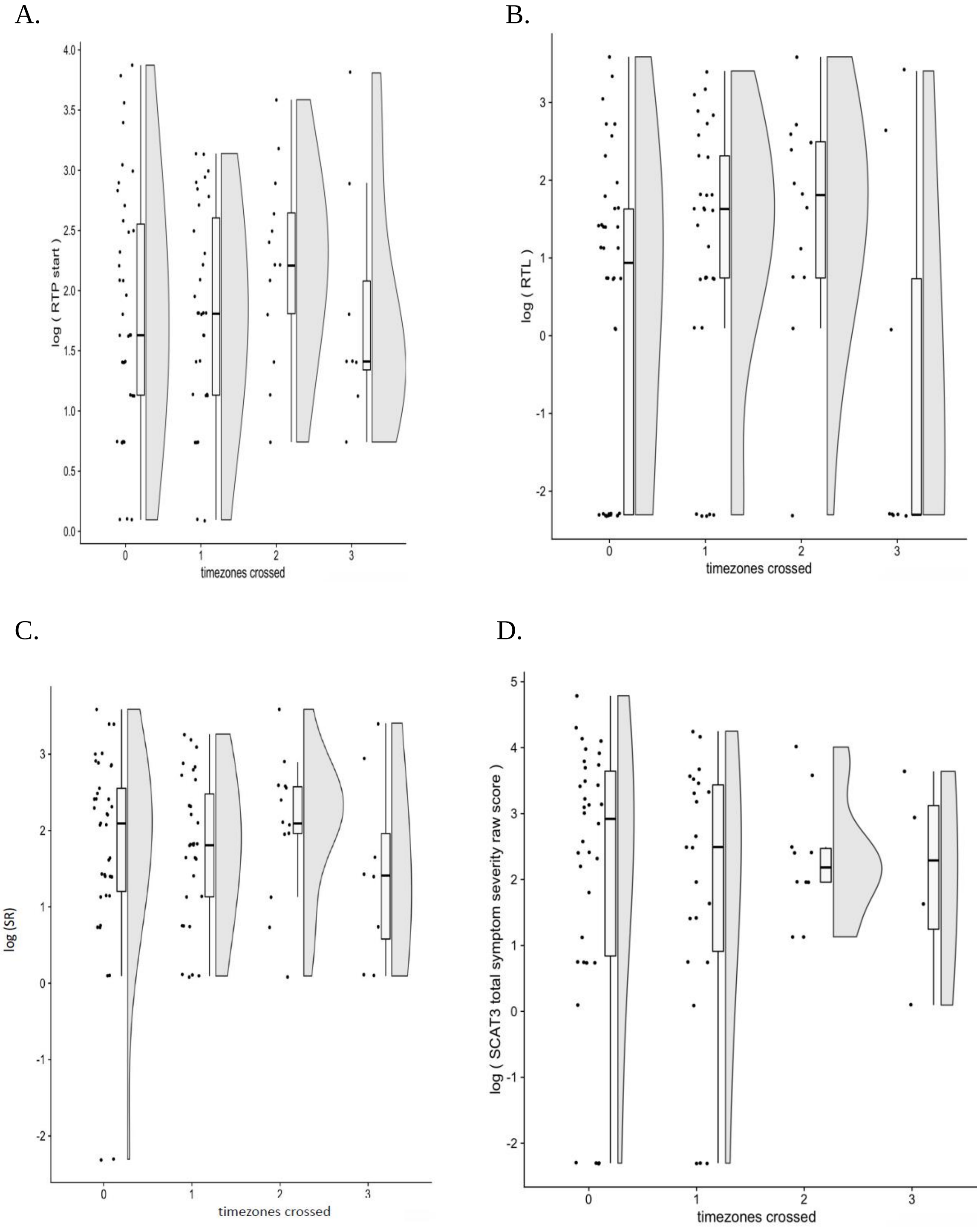

© 2020 Sharma TL et al. *JAMA Network Open.*

eFigure 6. Comparison of Symptom Recovery and Severity Outcome Variables and Time From Injury to Flight

See eFigure 3 for abbreviations (A: RTP start, B: RTL, C: SR, D: Symptom severity post-injury).

A.

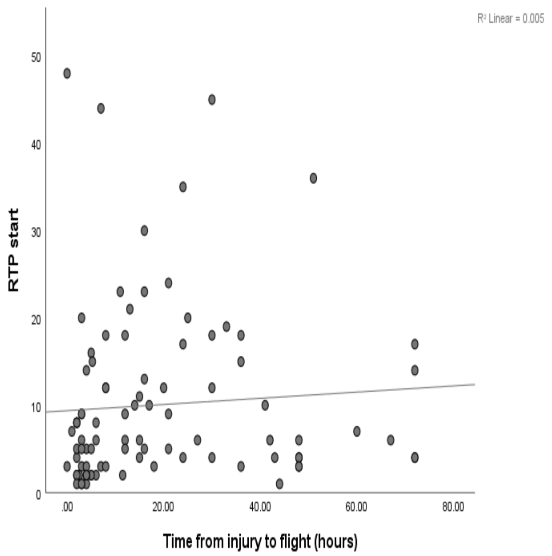

B.

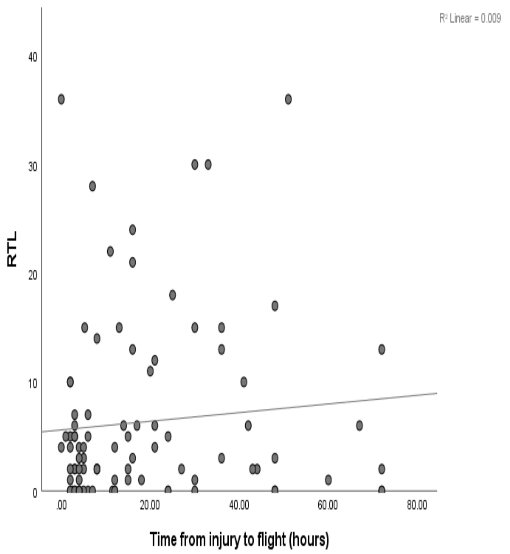

C.

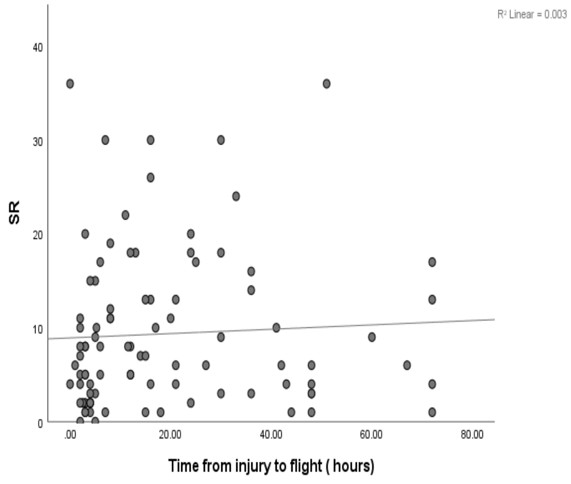

D.

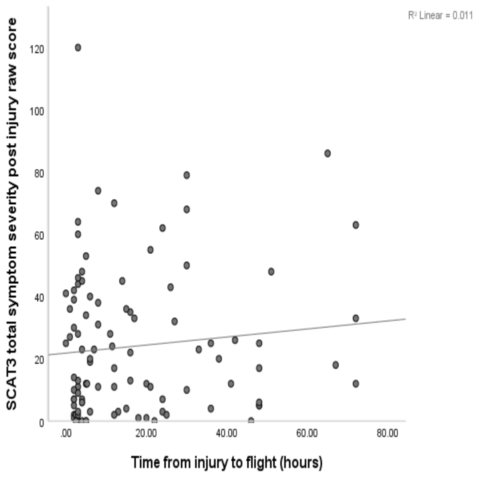

© 2020 Sharma TL et al. *JAMA Network Open.*
